# Unitization modulates recognition of within‐domain and cross‐domain associations: Evidence from event‐related potentials

**DOI:** 10.1111/psyp.13446

**Published:** 2019-08-01

**Authors:** Bingcan Li, Meng Han, Chunyan Guo, Roni Tibon

**Affiliations:** ^1^ Beijing Key Laboratory of Learning and Cognition, School of Psychology Capital Normal University Beijing China; ^2^ MRC Cognition & Brain Sciences Unit University of Cambridge Cambridge UK

**Keywords:** episodic memory, familiarity, recognition, recollection, unitization

## Abstract

Although it is often assumed that memory of episodic associations requires recollection, it has been suggested that, when stimuli are experienced as a unit, earlier memory processes might contribute to their subsequent associative recognition. We investigated the effects of associative relations and perceptual domain during episodic encoding on the ability to utilize early memory processes to retrieve associative information. During the study phase, participants encoded compound and noncompound words pairs, presented either to the same sensory modality (visual presentation) or to different sensory modalities (audiovisual presentation). At the test phase, they discriminated between old, rearranged, and new pairs while ERPs were recorded. In an early ERP component, differences related to associative memory emerged only for compounds, regardless of their encoding modality. These findings indicate that episodic retrieval of compound words can be supported by early‐onset recognition processes regardless of whether both words were presented to the same or different sensory modalities, and suggests that unitization can operate at an abstract level, across a broad range of materials.

## Abstract 2

We offer new insights into the multiplicity of processes that are associated with retrieval of unitized and ununitized associations. We used EEG while human participants learned and subsequently recognized compound and noncompound word pairs, presented to the same sensory modality or to different modalities. We were able to show that early associative recognition ERP effects only emerge for compounds, regardless their encoding modality. Our findings reinforce the importance of pre‐existing associative relationships to episodic remembering and suggest that unitization is capable of acting at an abstract level, across a broad range of materials.

## 1 INTRODUCTION

Remembering episodic associations—that several objects, people, or actions were experienced conjointly—is a core cognitive function. Such associations can involve a single sensory modality (the looks of the two people you've just met) or multiple sensory modalities (the sight and taste of a sandwich you've just ate); they can involve items that were already experienced together before (two of your friends from grad school sitting together) or items that are now experienced together for the first time (your friend from grad school walking down the street with your mother). Unitization—the creation of a new unit from individual items1In the current study, we adhere to the above definition of unitization, which is most commonly used in studies in the field. According to this definition, unitization is a process that binds together several items, thereby allowing interitem associations. Note, however, that in Mayes et al. (2007), unitization is alternatively defined as a process that binds together the various components of a single item, thus enabling the creation of intraitem associations.—has been proposed as an effective strategy for remembering episodic associations (e.g., Gobet et al., 2001; Graf & Schacter, 1989; Yonelinas, 1997; Yonelinas, Kroll, Dobbins, & Soltani, 1999), but the conditions that enable unitization require further specification. In the current study, we used EEG to examine the effect of different encoding conditions on unitization, as indicated by the time course of associative retrieval.

It is agreed that retrieval from episodic memory can be achieved via several distinct mnemonic processes that operate at different time points, and allow different levels of remembering. For example, the widely supported dual‐process theory of episodic memory posits that recognition tasks involve two separable processes: familiarity and recollection. Familiarity is a feeling of having encountered something or someone before, without retrieval of additional information, whereas recollection further provides contextual details about that encounter. This multiplicity of retrieval processes is supported by evidence from many behavioral, neurophysiological, and neuroimaging studies, including ERP studies showing that two qualitatively distinct ERP components are associated with recognition judgments. The first is the early midfrontal component, showing greater negative deflection for new versus old items, arising 300‒500 ms poststimulus presentation. This effect is traditionally described as the putative electrophysiological correlate of familiarity. The second is the late posterior component, showing greater positive deflection for old versus new items, and is prominent over left parietal electrodes 500‒800 ms poststimulus presentation. This effect is considered to be an electrophysiological correlate of recollection (reviewed by Mecklinger, 2000; Rugg & Curran, 2007; Wilding & Ranganath, 2011).

Notably, previous research has challenged the validity of the early midfrontal component as a direct index of familiarity. It has been argued that this electrophysiological component might reflect conceptual priming (Paller, Voss, & Boehm, 2007) or other “working‐with‐memory” operations (Moscovitch, 1992) such as pattern completion, which provide direct access to mnemonic representations (for discussion, see Tibon, Ben‐Zvi, & Levy, 2014; Tibon & Levy, 2014). Nevertheless, agreement remains that retrieval is accompanied by (at least) two electrophysiological components—an early midfrontal one, possibly reflecting familiarity, priming, or other forms of early mnemonic processes, and a late posterior one, reflecting strategic reconstruction via recollection.

While traditionally it has been asserted that recollection is required to retrieve episodic associations (e.g., Donaldson & Rugg, 1998, Hockley & Consoli, 1999, Yonelinas, 1997), in recent years, research has suggested that early retrieval processes, such as familiarity, can contribute to associative recognition under certain circumstances. Specifically, in a study by Quamme, Yonelinas, and Norman (2007), participants were given arbitrary word pairs such as CLOUD‐LAWN in the context of either a definition (e.g., “a garden used for sky gazing”) or a sentence (e.g., “the ______ could be seen from the ______”). It was presumed that the former, but not the latter, creates a unitized unit that allows the two words to be encoded as a compound. Strikingly, the study showed that, although amnestic patients with damage to the hippocampus and severe recollection deficits struggled to remember nonunitized pairs, their memory for unitized pairs was relatively intact, suggesting that memory for such pairs can rely on other mnemonic processes and does not require recollection. This notion was further supported by a growing body of evidence (for review, see Mecklinger & Jäger, 2009; Yonelinas, Aly, Wang, & Koen, 2010), showing that early retrieval processes are enabled when pairs of items are unitized (i.e., treated as a single unit rather than as a pairing of two separate items). Indeed, in addition to behavioral studies (e.g., Ahmad & Hockley, 2017; Diana, Yonelinas, & Ranganath, 2008; Parks & Yonelinas, 2015; Robey & Riggins, 2017; Shao, Opitz, Yang, & Weng, 2015; Tibon, Greve, & Henson, 2017; Tibon, Vakil, Goldstein, & Levy, 2012; Tu, Alty, & Diana, 2017) and fMRI studies (Bader, Opitz, Reith, & Mecklinger, 2014; Diana, Yonelinas, & Raganath, 2009; Ford, Verfaellie, & Giovanello, 2010; Haskins, Yonelinas, Quamme, & Ranganath, 2008; Memel & Ryan, 2017), electrophysiological studies have established that the early midfrontal ERP effect, which indicates early retrieval processes, is modulated following unitization encoding (e.g., Bader, Mecklinger, Hoppstädter, & Meyer, 2010; Kamp, Bader, & Mecklinger, 2016 [definition vs. sentence, as described above]; Diana, Van den Boom, Yonelinas, & Ranganath, 2011 [integral vs. contextual encoding of source information]; Guillaume & Etienne, 2015; Jäger, Mecklinger, & Kipp, 2006; Jäger, Mecklinger & Kliegel, 2010 [intrinsic vs. extrinsic facial information]; Rhodes & Donaldson, 2008 [following interactive imagery]; Tibon, Ben‐Zvi, & Levy, 2014 [unimodal vs. crossmodal stimulus pairs]; Tibon, Gronau, Scheuplein, Mecklinger, & Levy, 2014 [congruent vs. incongruent object pictures]; Zheng, Li, Xiao, Broster, & Jiang, 2015 [compound vs. unrelated word pairs]).

Two major theoretical frameworks account for mnemonic unitization effects. The first is the domain dichotomy (DD) view (Mayes, Montaldi, & Migo, 2007). This view extends earlier neurocognitive models of recognition memory, which associate the hippocampus with recollection and the perirhinal cortex with familiarity (for review, see Yonelinas, 2002) and proposes that the perirhinal cortex can mediate retrieval of intraitem and within‐domain associations (e.g., face–face associations), but that the hippocampus mediates retrieval of cross‐domain associations (e.g., scene–sound, face–voice, etc.), which require recollection. This distinction implies that early retrieval processes, such as familiarity, are limited to within‐domain associations, which can converge sufficiently within the perirhinal cortex. The second theoretical framework is the levels of unitization (LOU) account, which refers to the idea that there is a continuum along which associations can be unitized (Parks & Yonelinas, 2015). According to LOU, unitization is critically determined by the way in which individuals process the incoming stimuli (e.g., as a compound word or as two separate words). As such, it is assumed to operate at a fairly abstract level. Therefore, LOU predicts that unitization‐driven early retrieval processes should be available across different stimulus modalities or domains, such as words and faces or visual and auditory stimuli.

The discrepancy in the predictions posed by the two theoretical frameworks is also evident in empirical findings. Indeed, while some studies support the DD view, by showing that familiarity/nonhippocampal‐based retrieval is useful in supporting retrieval of within‐domain but not cross‐domain associations (Bastin, Van der Linden, Schnakers, Montaldi, & Mayes, 2010; Borders, Aly, Parks, & Yonelinas, 2017; Mayes et al., 2004; Tibon, Gronau et al., 2014; Troyer, D'Souza, Vandermorris, & Murphy, 2011; Vargha‐Khadem et al., 1997), others agree with the LOU account by showing no differences between within‐ and cross‐domain associations in nonhippocampal‐based retrieval (Park & Rugg, 2011; Turriziani, Fadda, Caltagirone, & Carlesimo, 2004) or even a reversed pattern (e.g., greater familiarity‐based retrieval of cross‐domain associations; Harlow, Mackenzie, & Donaldson, 2010; Parks & Yonelinas, 2015).

Parks and Yonelinas (2015) speculate that the complexity of the stimuli might be an important factor in determining why some but not other within‐domain stimuli would rely on perirhinal binding, and suggest that complex stimuli may be more difficult to unitize within domain because they impose greater attentional processing demands and/or because their combination does not result in a coherent unit. We propose that another possible source of this discrepancy is the nature of the associative relations between the to‐be‐unitized stimuli. As mentioned above, the LOU account suggests that stimulus pairs can vary along a continuum of their associative relations. On one end of this continuum are stimuli that already share strong associative relationships prior to the experiment, such as word compounds (e.g., “cottage pie” or “traffic jam”) or related object pictures presented in a coherent spatial configuration (e.g., a lamp over a desk). On the other end are stimuli that do not share any obvious relations prior to the experiment, such as semantically unrelated word pairs or object pictures. Crucially, the items comprising word compounds—stimuli that bear strong preexperimental associative relations—are often experienced via the same sensory modality. That is, in most cases, both words comprising a compound would be presented either visually or auditorily; cases where one word is presented visually and the other auditorily within the same event are extremely rare. Therefore, we propose that, when stimuli that hold preexisting relations are encoded within a domain, their relations are easily processed, possibly allowing within‐domain (but not cross‐domain) stimulus pairs to be bound within the perirhinal cortex.

The current study examined the effects of different encoding modes, that is, perceptual domain (within‐domain, cross‐domain) and word‐pair type (compound, noncompound) on the ability to create a unitized representation, subsequently remembered via early retrieval processes. Memoranda were either compound or noncompound word pairs, presented either visually or in an audiovisual presentation. During the study phase, participants performed a relatedness judgment for each word pair. At test, they discriminated old, rearranged, and new pairs while EEG was recorded. Both old and rearranged pairs were comprised of studied items. However, while old pairs further contain studied associative information, rearranged pairs contain novel associative information that was not presented during the study. Therefore, our main interest was in old/rearranged effects (i.e., differences between ERPs associated with correct old judgments vs. correct rearranged judgments, which are indicative of associative recognition). To ensure that item information is reinstated in both conditions, our design further included the new condition in order to capture trials with no memory for the items comprising the pairs. We assume that when the old/rearranged effect is apparent early on (in the time window and location associated with early retrieval processes), it indicates that stimuli were unitized at their encoding. Accordingly, we use the term “unitization effects” when referring to differences associated with the old/rearranged effect that occurs at frontal locations, during a time window of 300‒500 ms.

We set our experiment to test three main predictions. First, because compound words bear strong associative relationships and are thus located at the higher end of the unitization continuum (according to the LOU account), we predicted greater unitization effects for compounds than noncompounds. Second, with respect to unitization effects within/across perceptual domains, we predicted that if the DD view is correct, then greater unitization effects will emerge for within‐domain pairs, but that if the LOU account is correct, unitization effects will not differ for within‐domain and cross‐domain pairs. Third, given our proposal that preexisting relations are easily processed within domains but not across domains, allowing the former to benefit from perirhinal binding, we predicted that the difference in unitization effects for compounds versus noncompounds will be greater for within‐domain than for cross‐domain pairs. Furthermore, in addition to these specified, predefined examinations of unitization effects, we expanded our analyses to also examine other potential effects observed in the data. In particular, we aimed to identify the conditions, time windows, and locations in which ERP amplitude is associated with incremental reinstatement of mnemonic information and to examine whether the topography of associative recognition effects differs in the various conditions.

## 2 METHOD

### 2.1 Participants

Twenty right‐handed students from Capital Normal University were paid ¥30 per hour to take part in the study. Data from three participants were excluded due to insufficient artifact‐free trials in one or more conditions (*n* < 18) following EEG artifacts removal. Seventeen participants remained (11 women, mean age = 23.5, range = 20–26 years). All participants were native Chinese speakers, had normal or corrected‐to‐normal vision, and provided informed consent as approved by the Human Research Ethics Committee of Capital Normal University.

### 2.2 Stimuli

Study stimuli included a list of 600 Chinese word pairs, each comprised of two 2‐character words. The list was divided into two sublists: the first included 300 compounds (e.g., American movie) and the second included 300 noncompound word pairs with no associative or semantic relationship. The words in the two lists did not differ in their mean number of strokes (*M_compound_* = 16.5, *SD_compound_* = 4.57; *M_noncompound_* = 16.4, *SD_noncompound_* = 4.73; *t*(1198) = 0.36, *p* = .71) and in their mean frequency (occurrences per million, Liu, 1990; *M_compound_* = 56.15, *SD_compound_* = 97.07, *M_noncompound_* = 61.01, *SD_noncompound_* = 104.55; *t*(1198) = 0.83, *p* = .4).

To verify word assignment into sublists, we conducted a pilot study based on a protocol used in previous studies (Kriukova, Bridger, & Mecklinger, 2013; Rhodes & Donaldson, 2007; Zheng et al., 2015), in which 10 native Chinese speakers (7 women), who did not take part in the main experiment, were asked to judge how well the two words could be bound into a single concept using a scale from 1 (lowest ratings) to 7 (highest ratings). The results confirmed our initial assignment of word pairs into sublists and further showed that the set of compound word pairs received higher rating (mean = 5.94, *SD* = 0.86) than the set of noncompound word pairs (mean = 1.33, *SD* = 0.20), *t*(9) = 16.61, *p* < .0001).

**Figure 1:**
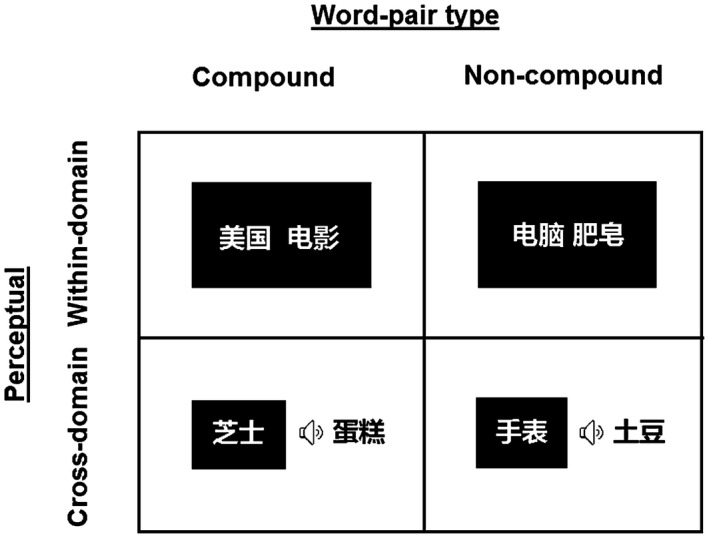
Example stimuli. Word‐pair type was either compound or noncompound. Perceptual domain was either visual for both words (within‐domain) or audiovisual (with one word presented visually and the other auditorily; cross‐domain). Examples of word pairs mean American movie (top left), cheese cake (bottom left), computer soap (top right), and watch potato (bottom right)

All word pairs were randomly assigned into 10 study‐test blocks, including five within‐domain blocks (visual‐visual) and five cross‐domain blocks (audiovisual; see Figure 1). Block order was interleaved and counterbalanced across participants (such that for half the participants the first block was a within‐domain block, and for the other half the first block was a cross‐domain block). Word pairs were matched across conditions, with equivalent number of strokes and word frequency. Auditory words were edited using Cool Edit software (mean length = 1,077 ms, *SD* = 185.7 ms).

In each block, the study phase consisted of 20 compounds and 20 noncompounds. In within‐domain blocks, both words were presented visually. In cross‐domain blocks, one word was presented visually, and the other was presented auditorily. The test phase of each block included 20 old word pairs (presented at study), 20 rearranged word pairs (constructed from words that were presented with other words at study), and 20 new word pairs. Importantly, old and rearranged words were presented at test with their perceptual domain (within‐domain, cross‐domain) unchanged from study. Additionally, rearranged pairs were presented at test with their type (compound, noncompound) unchanged from study, such that compounds were rearranged into new compounds and noncompounds were rearranged into new noncompounds. For example, the compound (e.g., 美国‐电影 meaning American movie) was rearranged into another compound (e.g., 美国‐总统 meaning American President) and the noncompound (e.g., 电脑‐肥皂 meaning computer soap) was rearranged into noncompound (e.g., 电脑‐空气 meaning computer air). Overall, the design included 12 conditions: 2 (Perceptual Domain: within‐domain, cross‐domain) × 2 (Word‐Pair Type: compound, noncompound) × 3 (Retrieval Category: old, rearranged, new), with 50 trials in each condition.

### 2.3 Procedure

Participants were seated in an electrically shielded and quiet room. Stimuli were displayed by Presentation software (Neurobehavioral Systems, San Francisco, CA) on a 17‐in. Dell computer monitor. Visual stimuli were displayed in white 18‐point Simhei font against a black background. Words were presented centrally (in the case of within‐domain pairs, the two words were presented side by side). At a viewing distance of 70 cm, the words subtended a maximum horizontal visual angle of 3.7° and a maximum vertical visual angle of 1.4°. Auditory words were read loudly and clearly by a male radio announcer and presented via earphones.

During the study phase, each trial began with the presentation of a fixation cross (+) at the center of the screen for 1,000 ms, after which the word pair was displayed. In within‐domain blocks, both words in the word pair were presented on the screen for 1,000 ms, followed by a blank screen for 500 ms. In cross‐domain blocks, one word was presented on the screen for 1,000 ms, with the auditory word concurrently presented through earphones. Participants were instructed to indicate whether the two words are related or not, by pressing marked keyboard keys using their left/right index finger. Key assignment was counterbalanced across participants.

During the test phase, each trial began with the presentation of a fixation cross for 1,000 ms. In within‐domain blocks, both words in the word pair were presented on the screen for 2,000 ms. In cross‐domain blocks, one word was presented on the screen for 2,000 ms, and the other was concurrently presented through earphones. During the test phase, participants were asked to judge whether the same word pair was presented during the study phase (old), whether the words comprising the pair were presented at study but with different pairing (rearranged), or whether the words comprising the word pair were not seen before (new). Participants provided their responses using the *F* key (with their left index finger), and the *J* and *K* keys (with their right index and middle finger). Hand assignment was counterbalanced across participants.

### 2.4 EEG data collection and preprocessing

EEG signals were recorded from 62 Ag/AgCl electrodes embedded in an elastic cap equipped with a NeuroScan SynAmps system and preprocessed with NeuroScan software. EEG data were collected at a sampling rate of 500 Hz with a 0.05–100 Hz band‐pass filter. Horizontal electro‐oculogram (EOG) were recorded bipolarly from electrodes placed 1 cm to the left and right of the outer canthi. Vertical EOG were recorded bipolarly from electrodes placed above and below the left eye. All voltages were referenced to the left mastoid online, and rereferenced offline to the average of the left and right mastoid. Impedance was less than 5 kΩ, and EEG/EOG signals were digitally band‐pass filtered from 0.05 Hz to 40 Hz. The EEG was segmented into 1,200‐ms epochs starting from 200 ms before the presentation of the stimuli and then subjected to baseline correction with the 200‐ms window preceding the stimuli. Blink artifacts were corrected using a linear regression estimate (Semlitsch, Anderer, Schuster, & Presslich, 1986). Subsequently, trials with voltage exceeding ±75 μV at any electrode and EEG artifacts other than blinks were rejected.

The EEG analyses included only trials with correct responses. Mean numbers of within‐domain analyzed trials were 45.4 (old), 32.1 (rearranged), and 40.1 (new) for compounds, and 36.4 (old), 34.9 (rearranged), and 39.8 (new) for noncompounds. Mean numbers of cross‐domain analyzed trials were 44.6 (old), 33.9 (rearranged), and 38.8 (new) for compounds, and 33.9 (old), 36.1 (rearranged), and 39.9 (new) for noncompounds. The minimal trial number in each condition was 20 (after excluding three participants who had fewer than 18 trials in one or more conditions, see Section 2.1 above).

### 2.5 Data analyses

For inferential statistics, repeated measures analyses of variance (ANOVAs) were conducted. Subsidiary analyses were performed using repeated measures ANOVAs or *t* tests as appropriate. Probability values (*p* values) for follow‐up analyses were adjusted by applying a Bonferroni correction. The significance level was set to α = .05. Given our interest in unitization effects on associative recognition, we focused mainly on differences between old and rearranged responses, although analyses and data associated with new responses are shown for completeness. Furthermore, because the current study focused on mnemonic effect, only main effects and interactions involving the factor of retrieval category are reported.

#### 2.5.1 Behavioral analyses

To analyze data from the study phase, repeated measures ANOVA was performed with factors of perceptual domain (within‐domain, cross‐domain) and word‐pair type (compound, noncompound) on relatedness rating (% related responses).

To examine memory performance during test, repeated measures ANOVA with factors of perceptual domain (within‐domain, cross‐domain), word‐pair type (compound, noncompound), and retrieval category (old, rearranged, new) was conducted on accuracy (% correct responses). To further analyze associative discrimination, associative Pr indices (the proportion of old pairs correctly classified as old minus the proportion of rearranged pairs incorrectly classified as old) were submitted to a repeated measures ANOVA, with perceptual domain and word‐pair type as within‐subject factors.

#### 2.5.2 ERP analyses

##### Predefined predictions

ERP analyses include two sections. The critical assumption underlying the current study was that unitized, but not ununitized, associations promote the contribution of early retrieval processes to associative recognition. Therefore, in the first section, we focused on the unitization effect (i.e., the early frontal old/rearranged effect, conventionally interpreted as the putative ERP correlate of early retrieval processes such as familiarity) to test the three predefined predictions specified in the Introduction: (a) that the unitization effect will be greater for compounds than noncompounds, (b) that the unitization effect will be greater for within‐domain than cross‐domain word pairs if the DD view is correct but will not differ between perceptual domains if the LOU account is correct, and (c) that the difference between compounds and noncompounds in the magnitude of the unitization effect will be greater for within‐domain than for cross‐domain pairs.

All three predictions were tested using a single repeated measures ANOVA, with perceptual domain (within‐domain, cross‐domain), word‐pair type (compound, noncompound), and retrieval category (old, rearranged) as repeated factors and with averaged data in the midfrontal site (Fz) in an early time window (300‒500 ms), where the early frontal effect is typically observed (reviewed by Friedman & Johnson, 2000; Mecklinger, 2000; Rugg & Curran, 2007; Wilding & Ranganath, 2011), as the dependent measure. For our first prediction, we expected a two‐way interaction between word‐pair type and retrieval category, such that the difference between old and rearranged responses (the unitization effect) would be greater for compounds than for noncompounds. For our second prediction, we expected a two‐way interaction between perceptual domain and retrieval category, such that the unitization effect would be greater for within‐domain than for cross‐domain pairs if the DD view is correct but not if the LOU account is correct. For our third prediction, we expected a three‐way interaction between all three factors, such that the difference between compounds and noncompounds in the magnitude of the unitization effect will be greater for within‐domain than for between‐domain word pairs. In addition, to allow interpretation of null results, we further tested the three predictions using Bayesian analyses, performed using R version 3.4.1 as implemented by RStudio version 1.0153, with a BayesFactor package (Morey & Rouder, 2015).

##### Exploratory analyses

**Figure 2:**
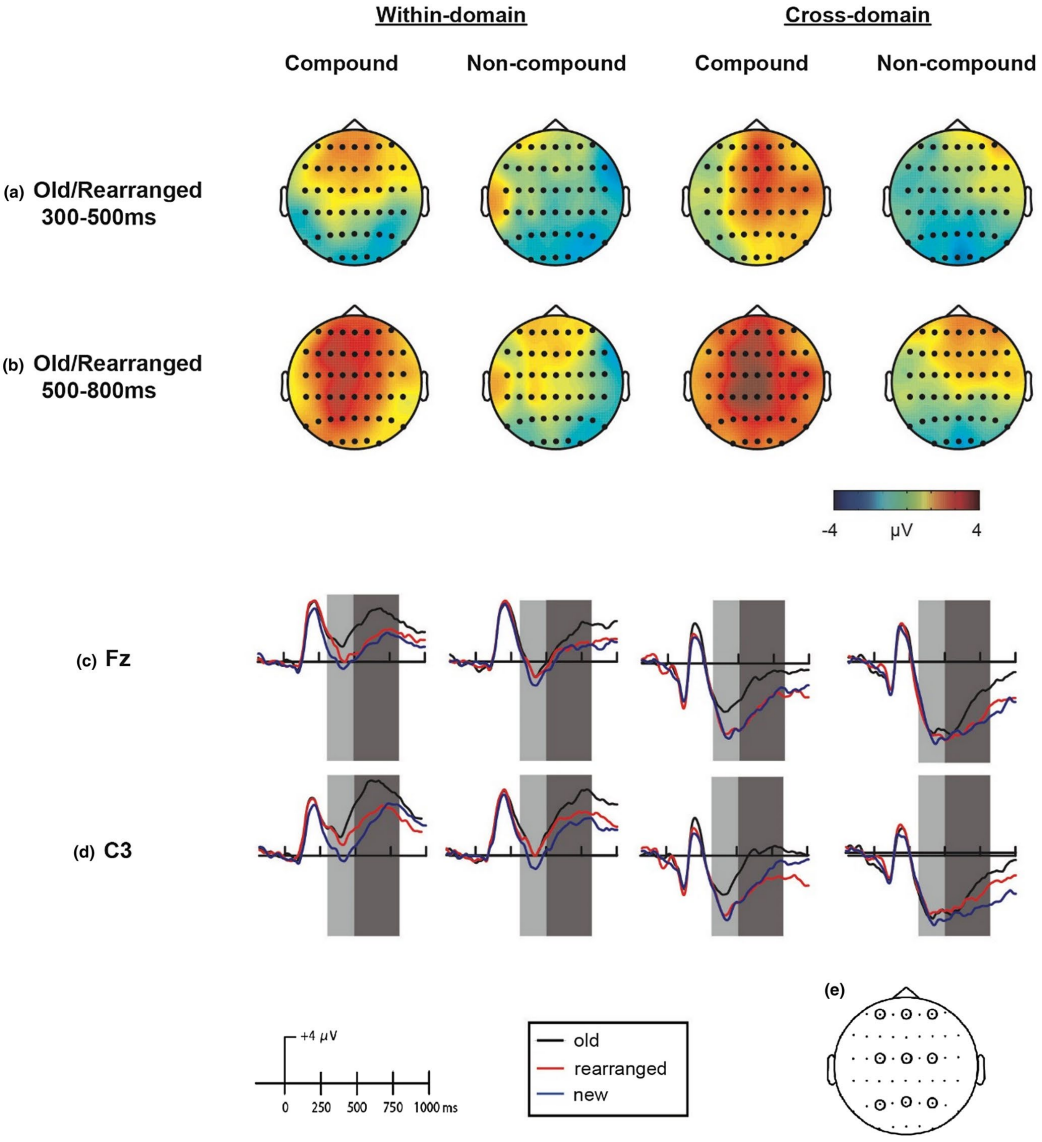
Topographic maps depicting the distribution of the old/rearranged effect in the (a) early (300‒500 ms), and (b) late (500‒800 ms) time windows, in the various perceptual domains (within‐domain, cross‐domain) and word‐pair types (compound, noncompound). ERP waveform for old (black), rearranged (red), and new (blue) responses in the various perceptual domains and word‐pair types at (c) Fz, and (d) C3. Analyzed time windows are highlighted in light gray (300–500 ms, early time window) and dark gray (500‒800 ms, late time window. (e) Schematic depiction of EEG channels implicated in the exploratory analyses: left frontal (F3), midfrontal (Fz), right frontal (F4), left central (C3), midcentral (Cz), right central (C4), left posterior (P3), midposterior (Pz), right posterior (P4)

In the second section of the ERP analyses, we provide additional analyses of the data that were not guided by specific predictions. First, although the main purpose of the new condition was to provide a clean measure of associative memory, the inclusion of this condition in the analyses can provide additional insights regarding more general retrieval processes. In particular, trials in the three retrieval categories represent incremental reinstatement of information during the test phase: for new word pairs, no episodic information is reinstated; for rearranged pairs, item information is reinstated; and for old pairs, both item and associative information are reinstated. To identify the conditions in which differences in ERP amplitude are associated with incremental reinstatement of mnemonic information, two standard time windows were selected, 300–500 ms (early) and 500–800 ms (late) to capture the early frontal and late posterior effects, respectively. Mean amplitudes for each condition were obtained from three frontal (F3, Fz, F4), three central (C3, Cz, C4), and three parietal (P3, Pz, P4) electrodes (see Figure 2e for a location map of analyzed electrodes). For each time window, we ran a repeated measures ANOVA on these mean amplitudes, with perceptual domain (within‐domain, cross‐domain), word‐pair type (compound, noncompound), retrieval category (old, rearranged, new), anteriority (anterior, central, posterior), and laterality (left, mid‐, right) as within‐subject factors. Significant interactions that included the factor of retrieval category were decomposed by fitting a linear contrast of old > rearranged > new across the various factors.

Second, we extended our analyses by examining topographical differences in the distribution of associative recognition effects using the entire montage of electrodes in both time windows. Differences in amplitude topography suggest that these effects might be mediated by distinct neural mechanisms (e.g., Allan, Robb, & Rugg, 2000). To directly compare topography of associative recognition effects in the different conditions, we first calculated the difference waves (old minus rearranged) for each participant for each condition. Difference amplitudes were then normalized according to the vector scaling procedure described by McCarthy and Wood (1985), applied within participants as was suggested by Haig, Gordon, and Hook (1997), and subjected to repeated measures ANOVA with perceptual domain (within‐domain, cross‐domain), word‐pair type (old, rearranged), and location (62 electrodes) as within‐subject factors.

## 3 RESULTS

### 3.1 Behavioral results

#### 3.1.1 Study phase

Data from the study phase are shown in Table 1. The analysis of this data revealed a main effect of word‐pair type, *F*(1, 16) = 2215.23, *p* < .0001, $\eta_{p}^{2}$ = .99, with increased proportion of related responses for compound than noncompound word pairs and a main effect of perceptual domain, *F*(1, 16) = 28.275, *p* < .0001, $\eta_{p}^{2}$ = .64, with increased proportion of related responses for within‐domain than cross‐domain word pairs.

**Table 1:** Related responses (%) in the various study conditions

Within‐domain		Cross‐domain	
Compound	Noncompound	Compound	Noncompound
97.41 (4.19)	10.59 (10.5)	95.65 (3.06)	3.88 (4.65)

Note

Standard deviations are shown in parentheses.

#### 3.1.2 Test phase: Accuracy

Table 2 shows accuracy data at test. The analysis of % accuracy revealed a main effect of word‐pair type, *F*(1, 16) = 6.86, *p* = .019, $\eta_{p}^{2}$ = .3, with greater accuracy for compounds compared to noncompounds and a main effect of retrieval category, *F*(2, 32) = 21.21, *p* < .0001, $\eta_{p}^{2}$ = .57, with greater accuracy for both old and new word pairs relative to rearranged word pairs (*p*s < .001). Furthermore, an interaction between perceptual domain and retrieval category was revealed, *F*(2, 32) = 3.43, *p* = .045, $\eta_{p}^{2}$ = .18, stemming from greater accuracy for cross‐domain compared to within‐domain word pairs for rearranged responses, *t*(16) = −2.74, *p* = .015, but not for old and new responses, *t*(16) = ‒0.9, *p* = .38; *t*(16) = .93, *p* = .47, respectively. In addition, an interaction between word‐pair type and retrieval category was revealed, *F*(2, 32) = 26.53, *p* < .0001, $\eta_{p}^{2}$ = .62, stemming from greater accuracy for compounds compared to noncompounds for old responses, *t*(16) = 5.9, *p* < .0001, no difference between compounds and noncompounds for new responses, *t*(16) = ‒0.38, *p* = .75, but marginally decreased accuracy for compounds compared to noncompounds for rearranged responses, *t*(16) = −1.95, *p* = .069.

**Table 2:** Mean accuracy (%) and associative Pr indices in the various test conditions

	Within‐domain		Cross‐domain	
	Compound	Noncompound	Compound	Noncompound
Old	95.61 (3.4)	75.76 (15.3)	95.53 (4.8)	73.53 (17.1)
Rearranged	67.47 (13.4)	72.85 (14.4)	72.82 (11.6)	78.47 (13.3)
New	84.0 (9.0)	82.91 (10.3)	83.65 (7.0)	86.24 (10.0)
**Associative Pr**	**63.61 (15.4)**	**48.62 (27.4)**	**68.35 (13.9)**	**52.00 (28.1)**

Note

Standard deviations are shown in parentheses.

Abbreviation: Pr, the proportion of old pairs correctly classified as old minus the proportion of rearranged pairs incorrectly classified as old.

For associative Prs (Table 2, bottom row), the analysis revealed a main effect for word‐pair type, *F*(1, 16) = 12.35, *p* = .003, $\eta_{p}^{2}$ = .44, with greater Pr values for compounds than for noncompounds and a marginal main effect of perceptual domain, *F*(1, 16) = 3.97, *p* = .06, $\eta_{p}^{2}$ = .2, with greater Pr values for cross‐domain than for within‐domain word pairs.

### 3.2 ERP results

#### 3.2.1 Predefined predictions

The ANOVA, which was set to test our predefined predictions, revealed a significant main effect of perceptual domain, *F*(1, 16) = 104.89, *p* < .0001, $\eta_{p}^{2}$ = .87, word‐pair type, *F*(1, 16) = 22.16, *p* < .0001, $\eta_{p}^{2}$ = .58, and retrieval category, *F*(1, 16) = 14.32, *p* = .002, $\eta_{p}^{2}$ = .47. Importantly, in accord with our first prediction, the analysis further revealed a significant interaction between word‐pair type and retrieval category, *F*(1, 16) = 5.05, *p* = .04, $\eta_{p}^{2}$ = .24, stemming, as predicted, from a greater unitization effect (i.e., early frontal old/rearranged effect) for compounds than noncompounds. However, the interaction between perceptual mode and retrieval category, predicted by the DD view (but not by the LOU account), was not significant, *F*(1, 16) = 0.53, *p* = .48, $\eta_{p}^{2}$ = .03. In addition, the three‐way interaction between these factors, specified by our third prediction, was not significant either, *F*(1, 16) = 0.69, *p* = .7, $\eta_{p}^{2}$ = .01.

Thus, the ANOVA analysis yielded null results for our second and third predictions. However, as with any form of classical null‐hypothesis testing, absence of evidence is not evidence of absence. We therefore used a Bayesian approach to compare null and alternate hypotheses. To test the first prediction, we collapsed across perceptual domains for each participant and subtracted the amplitude of rearranged responses from that of old responses, to calculate the magnitude of the old/rearranged effect separately for each word‐pair type. Then, we used a one‐sided Bayesian *t* test with a Cauchy prior scaled at sqrt(2)/2 (medium scaling), to compare the hypothesis that compounds elicit a greater old/rearranged effect compared to noncompounds (i.e., that the standardized effect size is bigger than 0, the so‐called alternative hypothesis) with the alternate null hypothesis, that the old/rearranged effect does not differ (i.e., that the standardized effect size is 0; the so‐called null hypothesis). Consistent with the ANOVA analysis, this analysis supported the alternative hypothesis, which was preferred by a Bayes factor of 3.48.

The same procedure was employed to test the second prediction, only this time we collapsed across word‐pair types, to calculate the magnitude of the old/rearranged effect separately for each perceptual domain. We compared two hypotheses: the LOU suggestion that the old/rearranged effect does not differ for within‐domain and cross‐domain word pairs and the DD suggestion that the old/rearranged effect is greater for within‐domain than cross‐domain word pairs. This analysis supported the former null hypothesis, which was preferred by a Bayes factor of 6.35. The data thus provide moderate evidence in support of the (null) prediction of the LOU account.

To test our third prediction, we calculated the old/rearranged effect for each participant in each condition and subtracted the magnitude of this effect for noncompounds from that of compounds, separately for each perceptual domain. We used the same Bayesian procedure described above, this time to compare the hypothesis that the difference between compounds and noncompounds in the magnitude of the old/rearranged effect does not differ for within‐domain and cross‐domain word pairs with the hypothesis that the old/rearranged effect for compounds versus noncompounds is greater for within‐domain than cross‐domain word pairs. This analysis also supported the null hypothesis, which was preferred by a Bayes factor of 5.26, thus providing moderate evidence against our third prediction.

#### 3.2.2 Exploratory analyses

Figure 2c,d shows grand‐mean ERPs for each condition, in a representative fontal electrode (Fz; where the associative recognition effect in the early time window was maximal) and a representative left‐central electrode (C3; where the effect in the late time window was maximal). Topographic plots of the difference between retrieval categories in the various conditions are included in the online supporting information. The results of the repeated measures ANOVAs, performed for the early and late time windows, are shown in Table 3. To further explore these effects, we decomposed significant interactions that included the retrieval category factor (Note: interactions decomposed in other analyses were not analyzed again in individual analyses) by examining the linear trend of old> rearranged> new across the various conditions.

**Table 3:** Outcomes of repeated measures ANOVAs for the early and late time windows

Time window	*df*	*F*	*p*	$\eta_{p}^{2}$
300‒500 ms				
Retrieval Category	2, 32	14.76	.000	.48
Retrieval Category × Anteriority	4, 64	12.43	.000	.44
Retrieval Category × Laterality	4, 64	6.04	.000	.27
Retrieval Category × Anteriority × Laterality	8, 128	2.40	.019	.13
Retrieval Category × Word Type	2, 32	6.93	.003	.30
Retrieval Category × Perceptual Domain	2, 32	3.75	.034	.19
Retrieval Category × Perceptual Domain × Anteriority × Laterality	8, 128	3.02	.005	.16
500‒800 ms				
Retrieval Category	2, 32	39.42	.000	.71
Retrieval Category × Anteriority	2.1, 33.9	3.62	.035	.18
Retrieval Category × Laterality	4, 64	7.24	.000	.31
Retrieval Category × Anteriority × Laterality	8, 128	3.68	.001	.19
Retrieval Category × Word Type	2, 32	11.57	.000	.42
Retrieval Category × Word Type × Anteriority	2.2, 35.7	4.07	.022	.20

Note

Only significant results that include the retrieval category factor are shown.

##### Early time window (300‒500 ms)

As shown in Table 3, top panel, in the early time window, a main effect of retrieval category was observed, adhering to a linear trend whereby ERPs for old word pairs were less negative than for rearranged word pairs, which were, in turn, less negative than for new word pairs. Decomposition of the interaction between retrieval category, anteriority, and laterality revealed that the linear trend was observed in all locations (all *p*s < .05), except for the left‐posterior channel, and was maximal at central and midline channels (*p*s < .001). Additionally, decomposition of the interaction between retrieval category and word‐pair type revealed that the linear trend was significant for compound words, *F*(1, 16) = 95.25, *p* < .001, $\eta_{p}^{2}$ = .86, but not for noncompounds. Finally, decomposition of the interaction between retrieval category, perceptual domain, anteriority, and laterality revealed that the linear trend was apparent in both perceptual domains (within‐domain: *F*(1, 16) = 27.16, *p* < .001, $\eta_{p}^{2}$ = .63; cross‐domain: *F*(1, 16) = 6.08, *p* = .025, $\eta_{p}^{2}$ = .28). However, for within‐domain pairs it was observed in all channels (all *p*s < .01), while for cross‐domain pairs it was only significant in two midline channels (Fz: *F*(1, 16) = 10.95, *p* = .036, $\eta_{p}^{2}$ = .41; Cz: *F*(1*,* 16) = 12.07, *p* = .027, $\eta_{p}^{2}$ = .43).

##### Late time window (500–800 ms)

As shown in Table 3, bottom panel, the linear trend of old> rearranged> new was also apparent in the late time window. Decomposition of the interaction between retrieval category, anteriority, and laterality revealed that the linear trend was observed in all locations (all *p*s < .01) and was maximal at left and frontal channels (*p*s < .001). Additionally, decomposition of the interaction between retrieval category, word‐pair type, and anteriority revealed that for compounds the linear trend was significant in all channels (all *p*s < .001), while for noncompounds it was significant only in central channels, *F*(1, 16) = 9.66, *p* = .021, $\eta_{p}^{2}$ = .38.

##### Topographic analyses

Figure 2a,b shows the topography of the difference between old and rearranged pairs in the various conditions (topographic plots of the difference between rearranged and new pairs and between old and new pairs are included in the supporting information). In the early time window, the topographic analysis revealed a significant main effect of location, *F*(6.6, 106.08) = 4.52, *p* < .001, $\eta_{p}^{2}$ = .22, but no other significant main effects or interactions. In the late time window, the analysis revealed a significant main effect of perceptual domain, *F*(1, 16) = 6.88, *p* = .018, $\eta_{p}^{2}$ = .3, and of location, *F*(6.55, 104.84) = 4.19, *p* < .001, $\eta_{p}^{2}$ = .21, as well as significant interactions between word‐pair type and location, *F*(6.32, 101.1) = 2.24, *p* = .042, $\eta_{p}^{2}$ = .12, and between perceptual domain, word‐pair type, and location, *F*(5.86, 93.8) = 2.49, *p* = .029, $\eta_{p}^{2}$ = .14.

## 4 DISCUSSION

In the current study, an associative recognition memory task was employed to explore whether episodic associations between compounds versus noncompounds and between within‐domain versus cross‐domain word pairs differentially recruit recognition‐related processes, as indicated by differences in the electrophysiological components associated with their retrieval. The critical assumption underlying our current study was that the ability of associations to be processed in a unitized fashion promotes the contribution of early retrieval processes to their associative recognition. Therefore, we focused on the early frontal old/rearranged effect but also evaluated other recognition effects in the early and late time windows. Our data provide novel evidence for a multiplicity of processes supporting associative recognition and afford new insights regarding two theoretical frameworks that account for mnemonic unitization effects—the DD view and the LOU account.

Our first key finding was that associative recognition of compounds (but not of noncompounds) was associated with modulation of an early frontal effect—conventionally interpreted as the putative electrophysiological correlate of early retrieval processes such as familiarity—as indicated by decreased frontal negativity for old compared to rearranged compounds in the early time window. Several previous electrophysiological studies (Bader et al., 2010; Diana et al., 2011; Guillaume & Etienne, 2015; Jäger et al., 2006, 2010; Kamp et al., 2016; Kriukova et al., 2013; Li, Mao, Wang, & Guo, 2017; Lyu, Wang, Mao, Li, & Guo, 2018; Rhodes & Donaldson, 2008; Tibon, Ben‐Zvi, & Levy, 2014; Tibon, Gronau et al., 2014; Zheng et al., 2015) also suggest that early retrieval processes are selectively enabled for unitized but not for nonunitized pair associates. However, only two prior electrophysiological investigations of unitization effects at the higher end of the LOU continuum (where stimuli already bear strong associative relationships prior to the experiment) employed a design that allows conclusive attribution of mnemonic effects to associative recognition (Kriukova et al., 2013, Zheng et al., 2015). Other studies do not afford direct examination of associative recognition effects, either because the experimental conditions in the design were not fully balanced (e.g., related stimuli were rearranged into unrelated retrieval pairs, Bader et al., 2010; Li et al., 2017; Rhodes & Donaldson, 2008; Tibon, Gronau et al., 2014; Wang, Mao, Li, Lu, & Guo, 2016) or because the analyses contrasted old/new responses, rather than old/rearranged responses (Bader et al., 2010; Rhodes & Donaldson, 2008; Wiegand, Bader, & Mecklinger, 2010). Notably, in such cases, the early frontal negativity can be interpreted in terms of semantic congruency rather than episodic memory (e.g., see discussion in Tibon, Cooper, & Greve, 2017) or in terms of item rather than associative recognition. Crucially, in the current study, old compounds were contrasted with rearranged compounds and old noncompounds were contrasted with rearranged noncompounds. Therefore, any retrieval effects can be attributed to the influence of a preexisting association on the *episodic* memory of the pairs (i.e., that the pair presented at test was encoded during the study phase). With this, our study joins a handful of previous electrophysiological studies (Kriukova et al., 2013, Zheng et al., 2015) by conclusively showing that strong associative relationships between stimuli prior to the experiment promote rapid episodic retrieval, indicated by an early onset of memory‐related differences in frontally distributed ERPs.

Our second key finding was that, in accord with the prediction of the LOU account and contrary to the prediction of the DD view, modulation of the early frontal effect by old versus rearranged pairs was not greater for within‐domain relative to cross‐domain associations. This was further evident in our Bayesian analysis, which preferred the null hypothesis of no difference. Our finding that within‐domain and cross‐domain associations equally modulate the early frontal effect (thereby suggesting that early mnemonic processes can become equally available for both types of perceptual domains), thus agreeing with the proposal of the LOU account that unitization operates at a fairly abstract level and is therefore also available across different stimulus modalities or domains.

Not only was there no unitization advantage for within‐domain word pairs, in the current study we observed a somewhat reversed pattern: our behavioral data revealed that accuracy (measured by associative Pr, see Table 2) was marginally better for cross‐domain than for within‐domain word pairs. This reversed pattern contrasts with some previous studies showing greater unitization advantage and nonhippocampal‐based retrieval for within‐domain associations (Bastin et al., 2010; Borders et al., 2017; Tibon, Ben‐Zvi, & Levy, 2014; Troyer et al., 2011; Vargha‐Khadem et al., 1997) but agrees with other studies showing that cross‐domain associations can also benefit from unitization (Harlow et al., 2010; Park & Rugg, 2011; Parks & Yonelinas, 2015; Turriziani et al., 2004). In the current study, we tested the hypothesis (formulated as our third prediction) that this discrepancy arises because the relation between stimuli presentation (within‐/cross‐domain) and unitization effects is mediated by preexisting associative relations between the stimuli, such that only stimuli that already bear a strong association (such as compound words) would benefit from a within‐domain presentation. Indeed, the results from the study phase of the experiment suggest that within‐domain trials were more likely to be judged as related compared to cross‐domain trials, thereby verifying our assumption that such relations are processed more easily within domain. Nevertheless, our study did not yield the predicted interaction between word‐pair type and perceptual domain on the magnitude of the unitization effect and therefore did not confirm the hypothesis that preexisting relations between the stimuli selectively promote unitization effects for within‐domain associations. An alternative hypothesis to the one we set to test in the current study is that relatively simple stimuli such as words are more easily unitized within domain compared to relatively complex stimuli such as fractals (Parks & Yonelinas, 2015). However, our current study does not support this hypothesis either, since the stimuli in our case were word pairs, which are considered by Parks and Yonelinas to be relatively simple. It is therefore yet to be determined why some studies, but not others, show greater unitization effects for within‐domain associations.

In addition to these key findings emerging from our predefined predictions, our exploratory data analyses further revealed incremental mnemonic effects in the early and late time windows. Importantly, in accord with our first key finding, in the early time window the linear trend of old> rearranged> new was absent for noncompound word pairs. Furthermore, the effect was more widely distributed for within‐domain versus cross‐domain word pairs, hinting to some early difference between these conditions that was not apparent in our focused analysis. This difference emerges from the inclusion of the new condition (rather than from the inclusion of additional channels, as the topography of the old/rearranged effect did not differ in the early time window—see results of the topographic analyses above) and possibly suggests that early retrieval processes can be more readily utilized to discriminate between unstudied and studied items presented to the same sensory modality, compared to different sensory modalities.

Unlike the early time window, in the late time window, the linear trend was observed for all experimental conditions. This indicates that, contrary to the early effect, which is only available for unitized associations (in our case—compound words), incremental reinstatement of episodic information via late retrieval processes is available for both unitized and nonunitized associations—as would be expected from an electrophysiological correlate of recollection. Nevertheless, the magnitude of the late effect differed across the various conditions and was greater for compounds versus noncompounds. This pattern is in accord with previous studies, which generally agree that congruent stimuli combinations, such as word compounds (vs. noncompounds), support high levels of processing (Craik & Lockhart, 1972), yielding rich and elaborate encoding. In turn, during retrieval, this elaborated encoding supports high levels of recollection (summarized by Craik, 2002), which in our case was presumably indicated by greater late positivity for old than rearranged pairs and for rearranged than new pairs.

Finally, the exploratory analyses further revealed that the conditions employed in the current study did not differ in their topographic distribution of the early old/rearranged effect but differed significantly in the distribution of the late old/rearranged effect. These results agree with the idea (e.g., Norman & O'Reilly, 2003) that, while early retrieval processes (familiarity) extract regularities to allow overlapping memory representations (thus arguably recruit similar neural sources), late retrieval processes (recollection) rely on pattern separation to allow distinct memory representations of similar inputs (and therefore might recruit varying neural sources, depending on the task at hand).

One potential limitation of the current study is that the manner of presentation in the different perceptual domains might have resulted in ancillary differences between the conditions. In particular, the simultaneous presentation of the two words might be perceived as nontypical in the auditory domain (where verbal signals unfold over time) but as typical in the visual domain, or otherwise might have resulted in increased semantic interference (e.g., Starreveld & La Heij, 1995) in the cross‐domain condition. It is therefore possible that interactions with the perceptual domain factor (such as the topography of old/rearranged effect in the late time window) reflect differential processes associated with nontypical materials or following semantic interference, rather than differences associated with the perceptual domain per se. Another limitation of the current study is that we only employed coarse behavioral measures (accuracy rates). This was done for practical reasons: any additional fine‐tuned behavioral measures, such as confidence ratings or remember/know responses, would have required further division of the trials into experimental bins, resulting in insufficient signal‐to‐noise ratio for the ERP measures. Consequently, however, our current design does not allow conclusive associations between the observed ERP components and more specific mnemonic processes. Nonetheless, our data clearly point to a neural distinction, whereby the contribution of the early ERP effect to associative recognition is limited to compounds but the contribution of the late ERP effect is available for all pair associates.

In summary, examining the electrophysiological correlates of episodic associative recognition, we report evidence suggesting that recognition of compounds amenable to unitization can be differentially supported by early onset associative recognition processes, regardless of whether both words comprising the compound were presented to the same sensory modality or to different sensory modalities. These results reinforce the importance of preexisting associative relationships between episodically associated memoranda, in determining how their co‐occurrence is experienced and subsequently remembered. Furthermore, our finding that unitization can support cross‐domain associations suggests that it can act as a useful encoding strategy across a broad range of experimental conditions and materials.

## Supporting information


**Figure S1**
