# Supplementary material for: Unitization modulates recognition of within‐domain and cross‐domain associations: Evidence from event‐related potentials
Source: Psychophysiology. 2019 Aug 1;56(11):e13446. doi: 10.1111/psyp.13446 (PMC6852485; doi:10.1111/psyp.13446)
Supplement: Supplementary file 1 — Figure S1 [file PSYP-56-na-s001.docx]

Topographic plots of the difference between retrieval categories in the various conditions

| ***300-500 ms*** | Within-domain | | Cross-domain | |
| --- | --- | --- | --- | --- |
|  | Compound | Non-compound | Compound | Non-compound |
| (A)  Old/Rearranged | 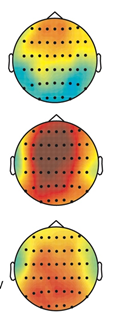 | 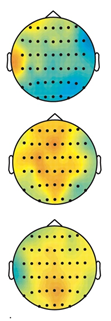 | 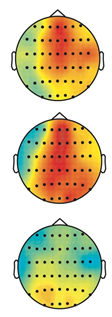 | 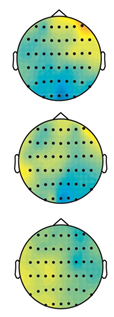 |
| (B)  Old/New |  |  |  |  |
| (C)  Rearranged/New |  |  |  |  |
|  |  |  |  | 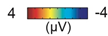 |

| ***500-800 ms*** | Within-domain | | Cross-domain | |
| --- | --- | --- | --- | --- |
|  | Compound | Non-compound | Compound | Non-compound |
| (A)  Old/Rearranged | 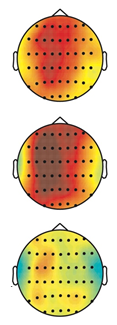 | 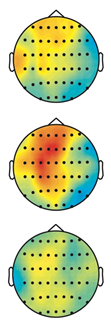 | 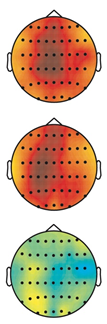 | 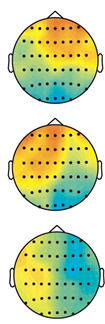 |
| (B)  Old/New |  |  |  |  |
| (C)  Rearranged/New |  |  |  |  |
|  |  |  |  | 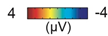 |
